# Baseline Sensitivity and Resistance Analysis of *Fusarium graminearum* to Pydiflumetofen in Henan Province, China

**DOI:** 10.3390/jof12030170

**Published:** 2026-02-27

**Authors:** Yun Wang, Dongmei Liu, Haiyan Yin, Cheng Cao, Yingni Cao, Dan Feng, Guanghua Zhao, Hongqi Wang, Jihong Liu

**Affiliations:** Institute of Agro-Product Quality and Safety, Henan Academy of Agricultural Sciences, Zhengzhou 450002, China

**Keywords:** *F. graminearum*, pydiflumetofen, sensitivity, succinate dehydrogenase, mutation

## Abstract

Fusarium head blight (FHB), caused by *Fusarium graminearum*, severely threatens wheat production in China’s Huang-Huai region. In order to clarify the resistance status of *F. graminearum* to pydiflumetofen in Henan Province, the mycelial growth rate method was used to assess the sensitivity of 345 strains isolated from 15 Henan cities during 2021–2024. The EC_50_ of *F. graminearum* isolates to pydiflumetofen was determined to be 0.016–3.981 μg/mL, with a right-skewed unimodal pattern, and the Shapiro–Wilk test confirmed a non-normal distribution (W = 0.4936, *p* < 0.05). Significantly higher mean EC_50_ values were observed in 2024 relative to 2021–2023, and resistant isolates were predominantly distributed in southwestern Henan Province. Fitness assays conducted in the absence of fungicide showed that most pydiflumetofen-resistant isolates exhibited similar mycelial growth, conidiation, pathogenicity, and deoxynivalenol (DON) production compared with sensitive isolates, suggesting no obvious fitness costs associated with pydiflumetofen resistance. Spearman rank correlation analysis demonstrated positive cross-resistance between pydiflumetofen and fluxapyroxad, but no cross-resistance to prothioconazole, phenamacril, or pyraclostrobin. Resistant strains had mutations in the FgsdhC2 (C89S, A93V) and FgsdhD (A21T, S30F) subunits of succinate dehydrogenase (SDH). Low-frequency pydiflumetofen resistance in *F. graminearum* from Henan Province highlights that pydiflumetofen should be applied alternately or in combination with fungicides showing no cross-resistance to delay the development of resistance.

## 1. Introduction

Fusarium head blight (FHB), caused by the *F. graminearum* species complex, is a devastating fungal disease. It not only reduces wheat yields but also contaminates grains with various mycotoxins such as deoxynivalenol (DON) and nivalenol (NIV), posing a severe threat to food safety and human health [1]. According to statistics from the Food and Agriculture Organization (FAO) of the United Nations, yield losses caused by epidemic FHB can reach as high as 10–20% in epidemic years [2]. As one of the world’s major wheat-producing countries, China has witnessed a gradual expansion of *F. graminearum* infestation from the middle and lower reaches of the Yangtze River wheat-growing region to the Huang-Huai wheat-growing region and further to northern and western wheat-growing areas in recent years. This expansion is driven by multiple factors including climate change, long-term straw returning practices, a simplified crop rotation system of wheat-maize monocropping, and adaptive evolution of the pathogen population, making *F. graminearum* a critical biological stress factor constraining national food security strategies [3,4].

Due to the lack of high-quality wheat varieties with strong resistance to FHB in practical production, chemical control remains the primary management strategy, characterized by its rapid efficacy, ease of operation, low cost, and remarkable control effects. At present, the control of fungal diseases in China mainly relies on four major classes of fungicides: benzimidazoles (e.g., carbendazim), demethylation inhibitors (DMIs) (e.g., tebuconazole, prothioconazole), methoxyacrylates (e.g., azoxystrobin, pyraclostrobin), and succinate dehydrogenase inhibitors (SDHIs, e.g., boscalid, fluopyram). However, most of these fungicides act on a single target site, which easily leads to the risk of resistance development. For instance, benzimidazoles such as carbendazim were once the mainstay fungicides for FHB control, but in recent years, the resistance level of field-collected strains to carbendazim has increased annually, with drug tolerance becoming increasingly prominent [5]. Chen et al. analyzed the resistance of 1118 *F. graminearum* strains from Henan Province to tebuconazole and found that 362 strains (accounting for 30.7%) exhibited resistance to this fungicide [6]. Although strobilurins possess broad fungicidal spectra and strong systemic properties, the agricultural application of cyazofamid faces a high risk of resistance development in *F. graminearum* populations [7]. Therefore, monitoring the resistance level of *F. graminearum* to pydiflumetofen is of great significance for implementing green control strategies against *F. graminearum* and reducing the application amount of chemical pesticides.

Pydiflumetofen belongs to the SDHI class of fungicides. Its mode of action involves specific binding to the ubiquinone-binding site composed of the SdhB, SdhC, and SdhD subunits of the succinate dehydrogenase complex. This binding blocks the electron transport chain of the tricarboxylic acid cycle (TCA), interfering with the energy metabolism of pathogenic fungi and thereby exerting a fungicidal effect [8]. With the large-scale agricultural application of SDHI fungicides, resistance development has been successively documented in various plant pathogenic fungi worldwide, with resistance mutation sites showing distinct diversity across different pathogens: Resistance mutations in the SdhB subunit have been confirmed in multiple fungal species. For example, homologous site mutations, including P225L/F/T, N230I, and H272L/R/Y in *Botrytis cinerea*; P225F, N230I, H272R, and H272Y in *Corynespora cassiicola*; and P226L in *Sclerotinia sclerotiorum*, have all been shown to confer resistance to pydiflumetofen and other SDHIs [9,10,11,12]. Mutations in the SdhC subunit also induce resistance, with reported examples including H151R in *Venturia inaequalis*, S73P in *Corynespora cassiicola*, and S73P and N75S in *Corynespora cassiicola* infecting cucumbers [11,13,14]. Resistance mutations in the SdhD subunit include H133R in *Alternaria solani* and D112E in *Septoria chrysanthemi* [15]. For *F. graminearum*, existing studies have clearly demonstrated that resistance development is directly associated with mutations in the SdhC subunit, among which mutations at sites such as R86H, R86C, A83V, and A73V are key factors leading to reduced sensitivity of the pathogen to this fungicide [16,17,18]. Nevertheless, current research on *F. graminearum* resistance to pydiflumetofen remains scarce, and critical issues such as the field occurrence dynamics of resistant strains have not yet been clarified. Therefore, resistance risk monitoring of *F. graminearum* to pydiflumetofen and elucidating the *Sdh* subunit mutation characteristics associated with resistance are of substantial theoretical and practical significance for formulating scientific anti-resistance management strategies and extending the field lifespan of pydiflumetofen.

## 2. Materials and Methods

### 2.1. Fungicides and Media

Technical-grade pydiflumetofen, prothioconazole, phenamacril, pyraclostrobin and fluxapyroxad (100 mg each) were provided by Ampuress Standard Technical Service Co., Ltd., Shanghai, China. Each technical grade was dissolved in acetone to prepare a stock solution with a concentration of 100 μg/mL, which was stored at 4 °C for further use.

Potato Dextrose Agar (PDA) medium was prepared as follows: 200 g of potato, 20 g of agar, and 20 g of glucose were dissolved in distilled water and made up to a final volume of 1 L, then autoclaved at 121 °C for 20 min before use.

Potato dextrose broth (PDB) medium was prepared with 200 g of peeled potatoes, 20 g of glucose, and 1000 mL of distilled water. The medium was autoclaved at 121 °C for 20 min prior to use.

### 2.2. Isolates and Culture of F. graminearum

A total of 345 *F. graminearum* strains were isolated from wheat scab-infected spikes collected from different regions in Henan Province during 2021–2024.

*F. graminearum* was isolated from symptomatic wheat spike tissues following the method of Nelson [19]. Briefly, small pieces (3–5 mm) from the margin of diseased lesions were surface-sterilized sequentially in 75% ethanol for 30–60 s, 5% sodium hypochlorite for 3–5 min, and rinsed 3–4 times with sterile distilled water. After drying on sterile filter paper, the tissues were placed onto PDA plates and incubated at 25 °C in the dark for 3–7 days. Pure cultures were obtained by repeated hyphal tip transfer on fresh PDA. Isolates were preliminarily identified based on colonial and microscopic morphological characteristics typical of *F. graminearum*. *Fusarium* identification was confirmed by species-specific PCR-based DNA analysis with the *Fg16F/Fg16R* primers using the method of Nicholson et al. [20]. Pure strains were maintained on PDA slants at 4 °C for short-term storage and in 20% glycerol at −80 °C for long-term preservation.

### 2.3. Establishment of Sensitivity Baseline to Pydiflumetofen

The discriminatory dose method [21] was employed to distinguish resistant isolates from sensitive ones. All 345 *F. graminearum* strains were inoculated onto PDA plates supplemented with 5 μg/mL pydiflumetofen and incubated at 25 °C in the dark for 3 days. The resistance phenotype of each strain was determined by mycelial growth on the amended medium. Strains showing no visible colony growth on the fungicide-amended plates were classified as sensitive strains (S), whereas strains capable of growing on the drug-containing medium were designated as resistant strains (R).

The mycelial growth rate method was adopted for sensitivity determination [22]. PDA media containing a series of pydiflumetofen concentrations (2.0, 1.5, 1.0, 0.6, 0.3, 0.1, and 0.01 μg/mL) were prepared. For each strain, 8 mm-diameter mycelial plugs were punched from the edge of 5-day-old pre-cultured colonies on PDA at 25 °C. The plugs were then inoculated onto PDA plates with different fungicide concentrations and onto blank PDA plates, with three replicates per concentration. After incubation at 25 °C in the dark for 4 days, colony diameters were measured using the cross method, and the mycelial growth inhibition rate was calculated. Toxicity regression analysis was performed using SPSS 22.0 software to determine the median effective concentration (EC_50_) and correlation coefficient. A sensitivity distribution diagram was constructed with the sensitivity interval of *F. graminearum* strains to pydiflumetofen as the abscissa and the strain distribution frequency as the ordinate.

### 2.4. Fitness Assessment and DON Content Determination of F. graminearum Strains in the Absence of Pydiflumetofen

Mycelial growth rate assay: Test strains were inoculated onto PDA plates and cultured at 25 °C for 3 days. Mycelial plugs of 8 mm diameter were excised from the colony edge and transferred to fresh PDA plates, followed by incubation at 25 °C in the dark for another 3 days. Colony diameters of all test strains were measured, with three replicates per treatment, and the entire experiment repeated three times.

Conidial production assay: Test strains were cultured on PDA plates at 25 °C for 3–4 days. Five 8 mm-diameter mycelial plugs were taken from the colony edge and inoculated into triangular flasks containing 30 mL of PDB medium. The flasks were incubated at 25 °C with shaking at 175 r/min in the dark for 5 days. Conidial yield was quantified using a hemocytometer under a microscope, with three flasks inoculated per strain, and the experiment repeated three times.

Pathogenicity assay: The susceptible wheat cultivar Yumai 49 was used for pathogenicity evaluation. Wheat kernels were soaked in distilled water for 12 h, placed in Petri dishes lined with moist filter paper, and germinated in a light incubator at 25 °C for 3 days, with periodic spraying of water to maintain humidity. Five 5 mm-diameter mycelial plugs were cut from the colony edge of each test strain and inoculated into triangular flasks containing 100 mL of PDB medium. After shaking incubation for 7 days, conidia were harvested and washed three times with sterile water. A conidial suspension with a concentration of 1 × 10^6^ conidia/mL was prepared using sterile water containing 0.01% Tween 20. The coleoptiles of germinated wheat seedlings were cut at 2–3 cm from the apex, and 5 μL of the conidial suspension was inoculated onto the cut surface (15 seedlings per strain). The inoculated seedlings were incubated in a light incubator at 25 °C for 15 days, after which the lesion length on the coleoptiles was measured. The experiment was repeated three times.

Determination of DON Content: The strains were cultured on PDA plates at 25 °C for 3 days. Mycelial plugs were taken from the colony margins and inoculated into 100 mL of PDB medium, followed by incubation in a shaker at 25 °C and 170 rpm for 7 days. After filtration, the culture filtrates were collected for the determination of DON content in different strains. A 5 mL aliquot of culture filtrate was transferred into a 50 mL centrifuge tube, and 20 mL of acetonitrile–water–acetic acid solution (70:29:1, *V*/*V*/*V*) was added. The mixture was vigorously vortexed at 2500 rpm for 1 min and then centrifuged at 8000 rpm for 5 min under refrigerated conditions. Exactly 750 μL of the supernatant was transferred to a 2 mL centrifuge tube, diluted with 750 μL of water, and mixed thoroughly. The mixture was centrifuged again at 11,000 rpm for 10 min under refrigeration, and then filtered through a 0.22 μm membrane filter into an autosampler vial. The samples were analyzed for DON content using liquid chromatography–mass spectrometry (LC–MS).

### 2.5. Determination of Cross-Resistance in F. graminearum

The sensitivities of 8 randomly selected sensitive isolates and 13 resistant isolates to prothioconazole, phenamacril, pyraclostrobin, and fluxapyroxad were determined using the mycelial growth rate method, with three replicates per treatment. Scatter plots were constructed using the log_10_(EC_50_) values of pydiflumetofen as the abscissa, and the log_10_(EC_50_) values of prothioconazole, phenamacril, pyraclostrobin, and fluxapyroxad as the ordinate, respectively. Cross-resistance between fungicides was evaluated using the Spearman rank correlation coefficient. *p* > 0.05 indicated no cross-resistance, whereas *p* < 0.05 indicated the presence of positive cross-resistance.

### 2.6. Sequence Analysis of Fgsdh Genes

Genomic DNA was extracted from both resistant and sensitive strains using the CTAB method. PCR amplification of the *FgSdh* genes was performed using the primer pairs designed by Chen et al. [21] (Table 1). The PCR reaction program was as follows: initial denaturation at 95 °C for 3 min; followed by 35 cycles of denaturation at 95 °C for 15 s, annealing at 56 °C for 15 s, and extension at 72 °C for 40 s; with a final extension step at 72 °C for 7 min. The PCR amplicons were sent to Sangon Biotech (Shanghai, China) Co., Ltd. for sequencing. The obtained sequences of *FgSdhA/B/C/D* genes were subjected to sequence alignment analysis using DNAMAN software (version 10.0).

### 2.7. Statistical Analysis

Statistical analysis was conducted using R software (version 4.4.3). Analysis of variance (ANOVA) followed by Tukey’s least significant difference (LSD) post hoc test (*p* < 0.05) was employed to identify significant differences among groups. All graphs were generated using R, and Spearman’s rank correlation analysis was performed to evaluate the cross-resistance relationship between pydiflumetofen and other fungicides.

## 3. Results

### 3.1. Sensitivity of F. graminearum to Pydiflumetofen in Henan Province

**Figure 1:**
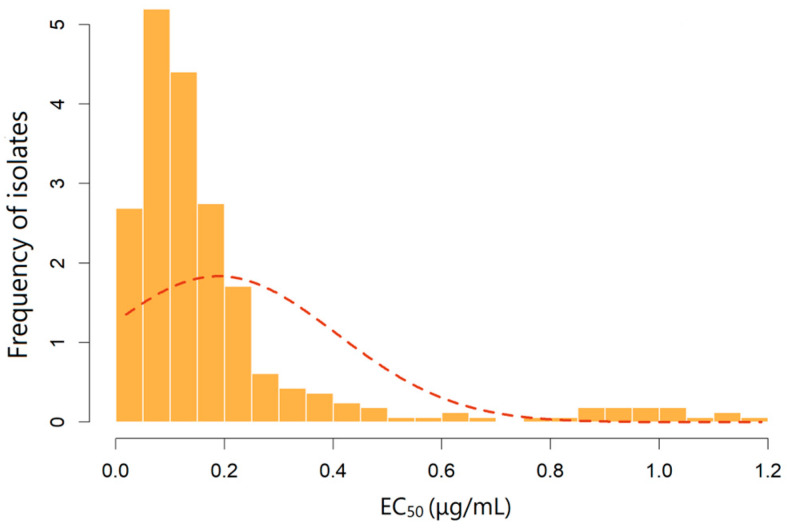
Sensitivity distribution frequency of *F. graminearum* to pydiflumetofen.

The EC_50_ values of 345 isolates of *F. graminearum* to pydiflumetofen ranged from 0.016 to 3.981 μg/mL, with a mean EC_50_ value of 0.289 ± 0.502 μg/mL (median: 0.101). The ratio of the maximum to minimum EC_50_ values was 248.8. The distribution of EC_50_ values was a distinctly right-skewed unimodal pattern, and a Shapiro–Wilk normality test confirmed that the data deviated significantly from a normal distribution (W = 0.4936, *p* < 0.05) (Figure 1), which is consistent with the emergence of field-resistant isolates.

A total of 25 pydiflumetofen-resistant isolates were detected among 345 wheat *F. graminearum* isolates, all of which were detected in isolates collected in 2024, with an overall resistance frequency of 7.25%. Specifically, 12 resistant isolates were found in Xinyang (resistance frequency = 3.48%), 4 each in Pingdingshan and Nanyang (1.16% each), 3 in Luoyang (0.87%), and 2 in Zhumadian (0.58%). No pydiflumetofen-resistant isolates were detected in the other 10 prefecture-level cities.

### 3.2. Differences in Sensitivity of F. graminearum to Pydiflumetofen Among Different Years

**Figure 2:**
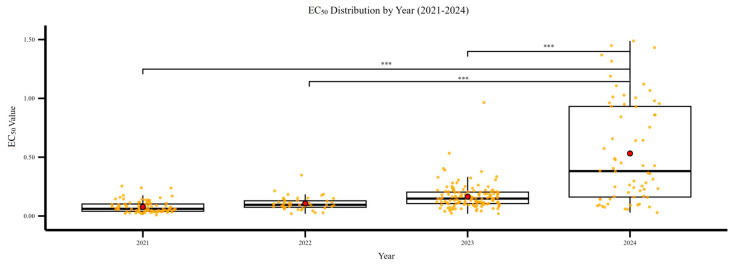
Sensitivity to pydiflumetofen of *F. graminearum* from 2021 to 2024. *** indicate significant differences at *p* < 0.05 by LSD test.

During the monitoring period, the sensitivity of *F. graminearum* to pydiflumetofen displayed a continuous decline, accompanied by a progressive expansion of intraspecific sensitivity variation. As shown in Figure 2, the mean EC_50_ value of isolates collected in 2024 was significantly higher than those from 2021 to 2023. The EC_50_ values of 2021–2023 isolates were mostly distributed within 0.01–0.2 μg/mL, with no obvious high-resistance individuals. In contrast, the 2024 population exhibited markedly increased dispersion of EC_50_ values, with numerous isolates exceeding 1.0 μg/mL and a maximum of 3.981 μg/mL. These high EC_50_ phenotypes formed a continuous gradient distribution, indicating that a resistant subpopulation had emerged and become established in the field by 2024, thus elevating the resistance risk of *F. graminearum* to pydiflumetofen.

### 3.3. Differences in Sensitivity of F. graminearum to Pydiflumetofen Among Different Regions

**Figure 3:**
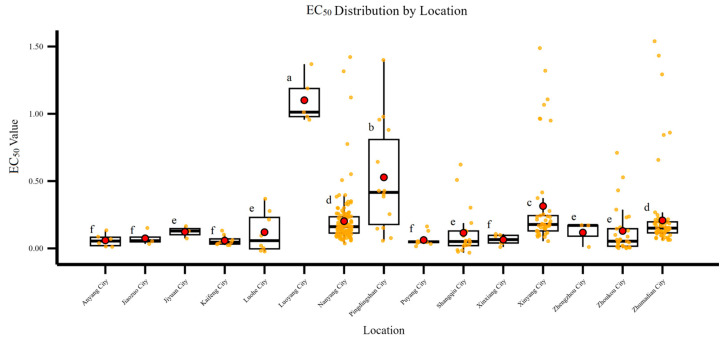
Sensitivity to pydiflumetofen of *F. graminearum* from different areas in Henan Province. Different letters indicate significant differences at *p* < 0.05 by LSD test.

As shown in Figure 3, significant differences in sensitivity to pydiflumetofen were observed among *F. graminearum* isolates from different geographical origins in Henan Province. Isolates from southwestern and southern Henan (e.g., Luoyang, Pingdingshan, Zhumadian, and Xinyang) exhibited significantly higher mean EC_50_ values than those from other regions. Regarding the dispersion of EC_50_ values, isolates from Nanyang, Zhumadian, and Xinyang showed the highest degree of sensitivity variation, with maximum EC_50_ values being 153-fold, 121-fold, and 88.6-fold higher than the corresponding minimum values, respectively, indicating a markedly dispersed distribution. In contrast, isolates from Kaifeng, Jiaozuo, Puyang, and Anyang were the most sensitive to pydiflumetofen, with the lowest degree of intraspecific sensitivity variation and a more concentrated EC_50_ distribution.

### 3.4. Fitness of Pydiflumetofen-Resistant and -Sensitive Strains of F. graminearum

Fitness analysis was performed on 13 randomly selected resistant isolates and 8 sensitive isolates. As shown in Table 2, significant differences were observed among different isolates in mycelial growth diameter, sporulation capacity, pathogenicity, and DON content, with no consistent pattern directly corresponding to the resistant or sensitive phenotype of the isolates. Notably, several resistant isolates exhibited superior biological characteristics. Among them, resistant isolate W24-051 produced a sporulation yield as high as 12.23 ± 0.26 × 10^5^ spores/mL, which was even higher than that of most sensitive isolates. This indicates that its sporulation ability was not significantly inhibited by resistance-related traits, suggesting strong reproductive fitness. In addition, resistant isolate W24-045 showed outstanding pathogenicity, with a lesion length of 1.29 ± 0.12 cm, exceeding the overall mean lesion length (0.95 cm) of all tested isolates. Its DON content was 1958.11 ± 149.85 μg/kg, at a moderate-to-high level, and its sporulation capacity was stable (5.09 ± 0.19 × 10^5^ spores/mL). Collectively, the fitness of this isolate was comparable to that of the sensitive isolates, implying a potential risk of spreading, reproducing, and becoming a dominant population in the field. Overall, the fitness of resistant isolates did not show a general declining trend, and some isolates displayed excellent pathogenicity and reproductive capacity.

### 3.5. Cross-Resistance Profile of F. graminearum to Pydiflumetofen, Prothioconazole and Phenamacril

**Figure 4:**
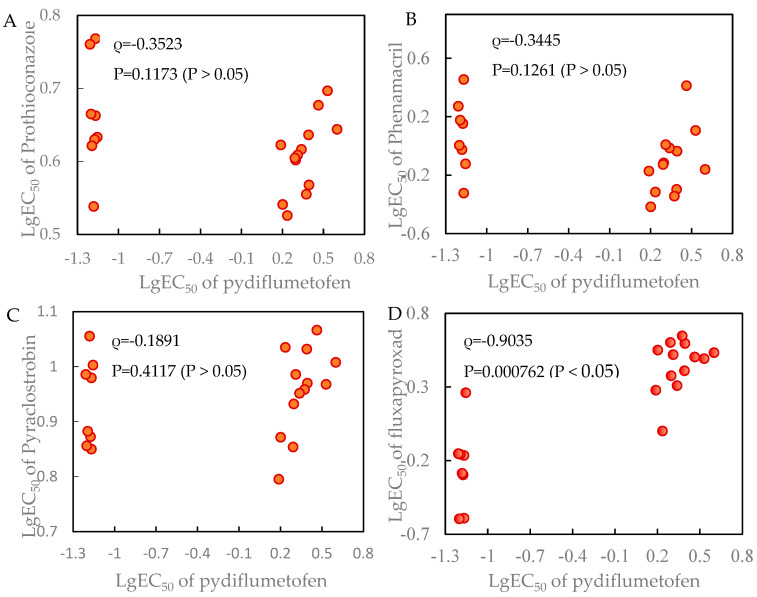
Cross-resistance between pydiflumetofen and other fungicides. (**A**) Prothioconazole; (**B**) Phenamacril; (**C**) Pyraclostrobin; (**D**) Fluxapyroxad. Spearman’s rank correlation coefficient was used to quantify cross-resistance.

Spearman rank correlation analysis of lgEC_50_ values among 21 *F. graminearum* isolates showed that pydiflumetofen was significantly correlated with fluxapyroxad, which also belongs to the SDHI class (ρ = 0.9035, *p* < 0.05), indicating positive cross-resistance (Figure 4). In contrast, no significant correlation was observed between pydiflumetofen and prothioconazole (ρ = −0.3523, *p* = 0.1173), phenamacril (ρ = −0.3445, *p* = 0.1261), or pyraclostrobin (ρ = 0.1891, *p* = 0.4117), suggesting no cross-resistance.

### 3.6. Sequence Analysis of Succinate Dehydrogenase (FgSdh A/B/C/D) Subunit Genes

**Figure 5:**
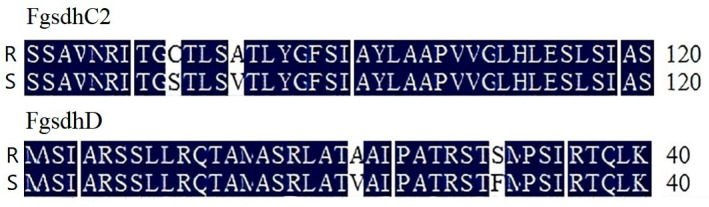
Partial Amino Acid Sequence analysis of FgSdhC2 and FgSdhD.

The target genes (*FgSdhB*, *SdhC1*, *FgSdhC2* and *FgSdhD*) of 13 resistant strains and 8 selected sensitive strains were cloned and sequenced. The results showed that no site mutations were detected in the DNA and amino acid sequences of *FgSdhA*, *FgSdhB* and *FgSdhC1* in all tested strains. In contrast, two amino acid substitutions were identified in both the FgSdhC2 and FgSdhD subunits of all resistant strains, and these mutations co-occurred in both subunits. Specifically, two distinct amino acid mutations were found in the FgSdhC2 subunit (Figure 5): cysteine (C) at position 89 was substituted with serine (S), and alanine (A) at position 93 was replaced by valine (V). Additionally, two amino acid mutations were detected in the FgSdhD subunit: alanine (A) at position 21 was substituted with threonine (T), and serine (S) at position 30 was replaced by phenylalanine (F).

## 4. Discussion

The evolution of pathogen resistance to fungicides is one of the primary factors limiting the efficacy of chemical control. Therefore, conducting regional resistance monitoring is of crucial scientific significance for the integrated management of plant diseases and the formulation of fungicide rotation strategies. To clarify the current sensitivity status of *F. graminearum* isolates from wheat in Henan Province to pydiflumetofen, the mycelial growth rate method was employed to determine their EC_50_ values. The results showed that the average EC_50_ of *F. graminearum* from major wheat-producing areas in Henan was 0.289 ± 0.502 μg/mL. The number of resistant isolates detected (25/345) was higher than those reported by Li et al. [23] in Hubei, Anhui, and Jiangsu Provinces of China (5/5157) and by Liu et al. [24] in a nationwide survey covering 67 counties (6/6468). This discrepancy may be attributed to the genetic differentiation of pathogen populations caused by differences in the application of SDHI fungicides and the wheat-maize rotation systems across different provinces.

Among the 345 *F. graminearum* isolates tested in this study, the EC_50_ values ranged from 0.016 to 3.981 μg/mL, indicating a significant variation in sensitivity to pydiflumetofen. The mean EC_50_ value of isolates collected in 2024 was significantly higher than those from other years, indicating a decrease in sensitivity to pydiflumetofen in the 2024 isolates. No significant difference in sensitivity to pydiflumetofen was observed among isolates from 2021 to 2023, although a gradual downward trend in sensitivity was detected during this period. This may be attributed to the sustained selection pressure imposed on the *F. graminearum* population by the continuous, single or frequent field application of pydiflumetofen since its introduction in 2021. Isolates from southwestern Henan (Luoyang, Pingdingshan, Nanyang, Zhumadian and Xinyang) showed significantly higher mean EC_50_ values for pydiflumetofen than those from other regions. In contrast, isolates from Kaifeng, Jiaozuo, Puyang and Anyang exhibited lower mean EC_50_ values, with no significant differences in sensitivity among isolates within these regions. This may be attributed to differences in field fungicide application practices and management.

Pydiflumetofen is a novel succinate dehydrogenase inhibitor (SDHI) fungicide, mainly used for the control of Fusarium diseases such as wheat head blight and crown rot [25]. Its production, registration and application shall comply with national pesticide management laws and regulations. Due to its single mode of action, this fungicide carries a high risk of resistance development. Relevant authorities have established specialized resistance management and application guidelines. Based on the regularity of pathogen resistance development and the resistance mechanism revealed in this study, it is necessary to optimize application strategies and establish an integrated management system combining chemical control and integrated pest management. To reduce selection pressure, this product shall not be used consecutively by itself. It should be applied in a mixture or rotation with fungicides of different modes of action and without cross-resistance. Registered pre-mix products follow the principle of complementary modes of action. Rotation schemes shall comply with the FRAC classification; consecutive use with other SDHI fungicides is prohibited, with an interval of at least one crop growth cycle.

The parasitic fitness of pathogens is a critical parameter for assessing their resistance risk. Previous studies have demonstrated no fitness costs in mycelial growth, sporulation, or virulence of resistant isolates [26,27]. In this study, eight pydiflumetofen-sensitive and thirteen resistant isolates were randomly selected for fitness analysis. The results indicated individual variations in mycelial growth rate, sporulation capacity, pathogenicity and DON content between resistant and sensitive isolates; no consistent fitness differences were observed between the tested resistant and sensitive strains, and some resistant strains still exhibited strong reproductive capacity, pathogenicity, and DON biosynthesis ability. These results indicate that resistant strains harboring such mutations maintain high fitness. The extensive application of pydiflumetofen may lead to the widespread establishment of resistant populations in the field, resulting in control failure. Therefore, it is necessary to strengthen the monitoring of resistant strains to determine their distribution frequency and resistance level in a timely manner.

Furthermore, to optimize the chemical control of wheat Fusarium head blight, this study investigated the cross-resistance between pydiflumetofen-resistant *F. graminearum* isolates and other fungicides. The results showed that there was positive cross-resistance between pydiflumetofen and fluxapyroxad, whereas no cross-resistance was observed with fungicides of other modes of action. These findings are consistent with the report by Zhou et al. [28], who demonstrated no cross-resistance between pydiflumetofen and seven other fungicide classes (tebuconazole, fluazinam, prochloraz, flutolanil, carbendazim, pyraclostrobin, or difenoconazole), and are also largely in agreement with the results of Miao et al. [29]. Thus, in regions with high incidence of Fusarium head blight and high levels/frequencies of SDHI resistance, the use of pydiflumetofen should be restricted, or it should be alternated with other fungicides or biocontrol agents with no observed cross-resistance to reduce the risk of field resistance development [30,31].

Resistance mutations to succinate dehydrogenase inhibitors (SDHIs) typically occur in the S*dhB*, S*dhC*, and S*dhD* subunits [32,33], with few reports of mutations in the *SdhA* subunit [34]. Previous studies on pydiflumetofen resistance in Fusarium species have also identified single amino acid mutations, including A83V or R86H/C substitutions in the SdhC subunit of *F. graminearum* [35], and H248Y (SdhB) and A64V or R67K (SdhC) substitutions in *Fusarium asiaticum* [21]. In this study, no mutations were detected in the *SdhA/B/C1* subunits of the tested isolates. However, mutations in the amino acid sequence of FgSdhC2 and FgSdhD subunits were detected in all resistant strains, and each of the two subunits had two mutation sites, namely C89S and A93V in the FgSdhC2 subunit, and A21T and S30F in the FgSdhD subunit. This differs from the FgSdhC1-A78V point mutation reported by Shao et al. [36] and the FpSdhC1- A83V/R86K mutations induced by Li et al. [37], suggesting that the mutations identified herein may arise from natural resistance evolution in the field. The detection of amino acid mutations in the FgSdhC2 subunit of resistant isolates is consistent with the conclusion by Duan et al. [38] that the *SdhC2* subunit regulates SDHI sensitivity in *F. asiaticum*. Although no *SdhC2* gene deletion was observed in resistant *F. graminearum* isolates, specific amino acid mutations (FgSdhC2-C89S/A93V) were detected. This finding further expands the mechanism underlying the role of the *SdhC2* subunit in resistance development [39]: naturally resistant isolates may alter the conformation of the SDH enzyme complex through amino acid substitutions at key subunit sites (rather than gene deletion), thereby reducing sensitivity to pydiflumetofen. However, it remains unclear whether this altered expression is the cause of the observed pydiflumetofen resistance or a consequence of the primary resistance mechanism. Therefore, further studies, including site-directed mutagenesis of each amino acid change (both individually and in combination), are required to evaluate their contributions to the resistance mechanism of the *F. graminearum* mutants identified in this study.

## 5. Conclusions

In summary, this study systematically revealed the current status of resistance to pydiflumetofen and the distribution characteristics of resistant isolates of *F*. *graminearum* in Henan Province. The results showed that the mean EC_50_ values of isolates collected in 2024 were significantly higher than those from 2021 to 2023, and the detected resistant isolates were mainly distributed in the southwestern region of Henan Province. Fitness assays revealed no consistent fitness costs in pydiflumetofen-resistant isolates compared with sensitive isolates, with no significant differences in mycelial growth, conidiation, pathogenicity, or DON production for most resistant isolates. Cross-resistance analysis indicated that pydiflumetofen exhibited cross-resistance with fluxapyroxad, but no cross-resistance with prothioconazole, phenamacril, or pyraclostrobin. Molecular detection revealed that resistant isolates harbored mutations in the succinate dehydrogenase subunits *FgSdhC2* (C89S, A93V) and *FgSdhD* (A21T, S30F). These findings provide a theoretical basis for the molecular monitoring of pydiflumetofen resistance in *F*. *graminearum* in the Huanghuai wheat region, and also offer important references for optimizing the resistance management system of SDHI fungicides and delaying the development of resistance.

## Figures and Tables

**Table 1:** Primer information for *SDH* gene amplification.

Primer Name	Sequence 5′–3′
*FgSdhA*-F	5′-ATGATGGCTTCTTCTATGG-3′
*FgSdhA*-R	5′-TTAGTATGTACGCTTGAAAG-3′
*FgSdhB*-F	5′-ATGGCTGCTCTCCGATCTTCCTC-3′
*FgSdhB*-R	5′-CTAGTTACCGAAAGCCATC-3′
*FgSdhC1*-F	5′-ATGTCACTGATCAATGTATT-3′
*FgSdhC1*-R	5′-CTACAACCAGAATGCCAATAC-3′
*FgSdhC2*-F	5′-ATGCTCGCTCAACGTGTTGG-3′
*FgSdhC2*-R	5′-TTACAGGAAAGCAACCAGACC-3′
*FgSdhD*-F	5′-ATGGCCTCAATTGCGCGTTC-3′
*FgSdhD*-R	5′-TTACGCCTTCCAGACTCGTC-3′

**Table 2:** Biological characters of *F. graminearum* strains with different sensitivities to pydiflumetofen.

Strain	Phenotype	Colony Diameter/cm	Sporulation/(10^5^ Spores/mL)	Lesion Size/cm
W23-183	S	6.35 ± 0.06 b	8.96 ± 0.16 b	1.27 ± 0.09 a
W24-027	S	6.82 ± 0.11 ab	12.09 ± 0.23 a	0.79 ± 0.07 bc
W24-031	S	7.21 ± 0.09 a	10.29 ± 0.20 ab	0.69 ± 0.10 c
W24-016	S	7.59 ± 0.12 a	9.87 ± 0.25 ab	1.06 ± 0.11 ab
W24-019	S	7.68 ± 0.06 a	7.67 ± 0.26 c	0.97 ± 0.09 b
W22-128	S	7.03 ± 0.05 ab	7.12 ± 0.27 c	0.96 ± 0.10 b
W23-317	S	7.32 ± 0.10 a	5.18 ± 0.15 d	1.32 ± 0.07 a
W23-293	S	6.97 ± 0.09 ab	6.74 ± 0.12 cd	0.85 ± 0.06 bc
W24-200	R	6.17 ± 0.12 b	7.21 ± 0.23 cd	0.67 ± 0.19 c
W24-242	R	7.83 ± 0.11 a	6.42 ± 0.25 cd	0.82 ± 0.15 bc
W24-034	R	7.77 ± 0.09 a	5.57 ± 0.15 d	0.59 ± 0.10 c
W24-045	R	7.31 ± 0.12 a	5.09 ± 0.19 d	1.29 ± 0.12 a
W24-141	R	6.56 ± 0.12 ab	11.25 ± 0.31 a	0.72 ± 0.11 c
W24-039	R	7.23 ± 0.14 a	9.89 ± 0.28 b	1.09 ± 0.15 ab
W24-041	R	7.26 ± 0.13 a	10.37 ± 0.16 ab	0.87 ± 0.12 bc
W24-138	R	6.97 ± 0.10 ab	8.82 ± 0.12 bc	1.13 ± 0.10 a
W24-040	R	7.52 ± 0.15 a	9.31 ± 0.21 b	0.96 ± 0.13 b
W24-139	R	7.16 ± 0.13 ab	7.95 ± 0.13 c	0.92 ± 0.09 b
W24-051	R	6.12 ± 0.10 b	12.23 ± 0.26 a	1.26 ± 0.17 a
W24-046	R	6.87 ± 0.13 ab	10.92 ± 0.18 ab	0.78 ± 0.12 c
W24-197	R	7.39 ± 0.17 a	9.13 ± 0.09 b	0.99 ± 0.08 b

Different letters in the same column indicate significant differences at *p* < 0.05 by LSD test.

## Data Availability

The original contributions presented in this study are included in the article. Further inquiries can be directed to the corresponding authors.

## References

[1] Xiu Q., Bi L.Y., Xu H.R., Li T., Zhou Z.H., Li Z.K., Wang J.X., Duan Y.B., Zhou M.G. (2021). Antifungal activity of quinofumelin against *Fusarium graminearum* and its inhibitory effect on DON biosynthesis. Toxins.

[2] Ma H.X., Zhang X., Yao J.B., Cheng S.H. (2019). Breeding for the resistance to *Fusarium* head blight of wheat in China. Front. Agric. Sci. Eng..

[3] Savary S., Willocquet L., Pethybridge S.J., Esker P., McRoberts N., Nelson A. (2019). The global burden of pathogens and pests on major food crops. Nat. Ecol. Evol..

[4] Zhou H.F., He X.L., Wang S., Ma Q.Z., Sun B.J., Ding S.L., Chen L.L., Zhang M., Li H.L. (2019). Diversity of the *Fusarium* pathogens associated with crown rot in the Huanghuai wheat-growing region of China. Environ. Microbiol..

[5] Qiu J.B., Huang T.T., Xu J.Q., Bi C.W., Chen C.J., Zhou M.G. (2012). *β-Tubulins* in *Gibberella zeae*: Their characterization and contribution to carbendazim resistance. Pest. Manag. Sci..

[6] Chen J.P., Wei J.Q., Fu L.Y., Wang S., Liu J.L., Guo Q.W., Jiang J., Tian Y., Che Z.P., Chen G.Q. (2021). Tebuconazole resistance of *Fusarium graminearum* field populations from wheat in Henan Province. J. Phytopathol..

[7] Chen Y., Zhou M.G. (2009). Characterization of *Fusarium graminearum* Isolates Resistant to Both Carbendazim and a New Fungicide JS399-19. Phytopathology.

[8] Zhang C., Liu Z.Y., Yang Y.G., Ma Q.H., Zheng Y.X., Xu C.X., Gao X.H., Gao W.N., Huang Z.Q., Liu X.L. (2024). Characterization of *Fusarium* species causing soybean root rot in Heilongjiang, China, and mechanism underlying the differences in sensitivity to DMI fungicides. Pestic. Biochem. Physiol..

[9] Ma J.h., Ying M.X., Lu Z.W., Guan Z.W., Zhang C.Q., Zhu X.L., Yang G.F. (2025). The resistance mechanism of B_P225F and B_H272R mutations in succinate dehydrogenase in *Botrytis cinerea*. Int. J. Biol. Macromol..

[10] Veloukas T., Leroch M., Hahn M., Karaoglanidis G.S. (2011). Detection and molecular characterization of boscalid-resistant *Botrytis cinerea* isolates from strawberry. Plant Dis..

[11] Zhu F.D., Shi Y.X., Xie X.W., Cha A.L., Li B.J. (2019). Occurrence, distribution, and characteristics of boscalid-resistant Corynespora cassiicola in China. Plant Dis..

[12] Wang Q., Mao Y.S., Li S.X., Li T., Wang J.X., Zhou M.G., Duan Y.B. (2022). Molecular mechanism of *Sclerotinia sclerotiorum* resistance to succinate dehydrogenase inhibitor fungicides. J. Agric. Food Chem..

[13] Ayer K.M., Villani S.M., Choi M.W., Cox K.D. (2019). Characterization of the *VisdhC* and *VisdhD* genes in *Venturia inaequalis*, and sensitivity to fluxapyroxad, pydiflumetofen, inpyrfluxam, and benzovindiflupyr. Plant Dis..

[14] Duan Y.B., Xin W.J., Lu F., Li T., Li M.X., Wu J., Wang J.X., Zhou M.G. (2019). Benzimidazole and QoI resistance in *Corynespora cassiicola* populations from greenhouse cultivated cucumber: An emerging problem in China. Pestic. Biochem. Physiol..

[15] Avenot H.f., Michailides T.J. (2010). Progress in understanding molecular mechanisms and evolution of resistance to succinate dehydrogenase inhibiting (SDHI) fungicides in phytopathogenic fungi. Crop Prot..

[16] Sun H.Y., Cui J.H., Tian B.H., Cao S.L., Zhang X.X., Chen H.G. (2020). Resistance risk assessment for *Fusarium graminearum* to pydiflumetofen, a new succinate dehydrogenase inhibitor. Pest Manag. Sci..

[17] Jiang J., Hu B.Y., Gao X.H., Cui Y.Y., Qian L., Xu J.Q., Liu S.M. (2025). In vitro determination of the sensitivity of *Fusarium graminearum* to fungicide fluopyram and investigation of the resistance mechanism. J. Agric. Food Chem..

[18] Sun H.Y., Cai S.Y., Liu H.Q., Li X.L., Deng Y.Y., Yang X.Y., Cao S.L., Li W., Chen H.G. (2023). FgSdhC paralog confers natural resistance toward SDHI fungicides in *Fusarium graminearum*. J. Agric. Food Chem..

[19] Nelson P.E. (1983). Fusarium species. An Illustrated Manual for Identification.

[20] Nicholson P., Simpson D.R., Weston G., Rezanoor H.N., Lees A.K., Parry D.W., Joyce D. (1998). Detection and quantification of *Fusarium culmorum* and *Fusarium graminearum* in cereals using PCR assays. Physiol. Mol. Plant Pathol..

[21] Chen W.C., Wei L.L., Zhao W.C., Wang B.R., Zheng H.H., Zhang P.C., Lou T.C., Duan Y.B., Hou Y.P., Zhou M.G. (2021). Resistance risk assessment for a novel succinate dehydrogenase inhibitor pydiflumetofen in *Fusarium asiaticum*. Pest Manag. Sci..

[22] Liu S.M., Che Z.P., Chen G.Q. (2016). Multiple-fungicide resistance to carbendazim, diethofencarb, procymidone, and pyrimethanil in field isolates of *Botrytis cinerea* from tomato in Henan Province, China. Crop Prot..

[23] Li Y.G., Zhang Z.Y., Yin X.R., Guo Y., Meng X.H., Zhang J., Cai Y.Q., Sheng G.L., Zhou M.G., Yuan S.K. (2025). A83V Mutation in FaSDHC2 Confers High Field Resistance to Pydiflumetofen and Exhibits Negative Cross-Resistance to Benzovindiflupyr in *Fusarium asiaticum*. J. Agric. Food Chem..

[24] Liu C., Shao W.Y., Duan Y.B., Zhao Y.F., Liu Z.Y., Ma Z.H. (2024). Biological and molecular characterization of pydiflumetofen and phenamacril dual-resistant *Fusarium graminearum* strains. Pest Manag. Sci..

[25] Dooley H., Shaw M., Spink J., Kildea S. (2016). The effect of succinate dehydrogenase inhibitor/azole mixtures on selection of Zymoseptoria tritici isolates with reduced sensitivity. Pest Manag. Sci..

[26] Li Y.W., Tang Y.D., Xue Z.W., Wang Y., Shi Y.F., Gao X.H., Li X., Li G.X., Li F., Lu L. (2023). Resistance Risk and Resistance-Related Point Mutation in SdhB and SdhC1 of Cyclobutrifluram in *Fusarium pseudograminearum*. J. Agric. Food Chem..

[27] Chen S.Q., Wu L.T., Zhao Z., Qi Z.Q. (2024). Baseline sensitivity and resistance analysis of *Botrytis cinerea* to pydifumetofen in Liaoning Province, China. Eur. J. Plant Pathol..

[28] Zhou F., Zhou H.H., Han A.H., Guo K.Y., Liu T.C., Wu Y.B., Hu H.Y., Li C.W. (2022). Mechanism of pydiflumetofen resistance in *Fusarium graminearum* in China. J. Fungi.

[29] Miao J.Q., Li Y.W., Hu Y.W., Li G.X., Gao X.H., Dai T., Liu X.L. (2024). Resistance risk, resistance mechanism and the effect on DON production of a new SDHI fungicide cyclobutrifluram in *Fusarium graminearum*. Pestic. Biochem. Physiol..

[30] Samaras A., Hadjipetrou C., Karaoglanidis G. (2021). Bacillus amyloliquefaciens strain QST713 may contribute to the management of SDHI resistance in *Botrytis cinerea*. Pest Manag. Sci..

[31] He L., Cui K., Song Y., Li T., Liu N., Mu W., Liu F. (2020). Activity of the novel succinate dehydrogenase inhibitor fungicide pydifumetofen against SDHI-sensitive and resistant isolates of *Botrytis cinerea* and efcacy against gray mold. Plant Dis..

[32] Sun H.Y., Lu C.Q., Li W., Deng Y.Y., Chen H.G. (2017). Homozygous and heterozygous point mutations in succinate dehydrogenase subunits b, c and d of *Rhizoctonia cerealis* conferring resistance to thifluzamide. Pest Manag. Sci..

[33] Avenot H.F., Sellam A., Michailides T.J. (2010). Characterization of mutations in the membrane anchored subunits of AaSDHC and AaSDHD of succinate dehydrogenase from *Alternaria alternata* isolates conferring field resistance to the fungicide boscalid. Plant Pathol..

[34] Lee J., Elliott M., Kim M., Yamada T., Jung G. (2021). A rapid molecular detection system for SdhB and SdhC point mutations conferring differential SDHI resistance in Clarireedia populations. Plant Dis..

[35] Zhang J., Sun S.Y., Guo Y., Ren F.H., Sheng G.L., Wu H., Zhao B.Q., Cai Y.Q., Gu C.Y., Duan Y.B. (2025). Risk assessment and resistant mechanism of *Fusarium graminearum* to fluopyram. Pestic. Biochem. Physiol..

[36] Shao W.Y., Wang J.H., Wang H.Y., Wen Z.Y., Liu C., Zhang Y., Zhao Y.F., Ma Z.H. (2022). *Fusarium graminearum* FgSdhC1 point mutation A78V confers resistance to the succinate dehydrogenase inhibitor pydiflumetofen. Pest Manag. Sci..

[37] Li Y.W., Wang Y., Li X.Y., Fan H.J., Gao X.H., Peng Q., Li F., Lu L., Miao J.Q. (2023). Resistant risk and resistance-related point mutation in SdhC1 of pydiflumetofen in *Fusarium pseudograminearum*. Pest Manag. Sci..

[38] Duan Y.B., Li M.X., Zhao H.H., Lu F., Wang J.X., Zhou M.G. (2018). Molecular and biological characteristics of laboratory metconazole-resistant mutants in *Fusarium graminearum*. Pestic. Biochem. Physiol..

[39] Xue Z.L., Zhong S., Shen J.H., Sun Y., Gao X.S., Wang X.Y., Li F., Lu L., Liu X.L. (2023). Multiple mutations in SDHB and SDHC2 subunits confer resistance to the succinate dehydrogenase inhibitor cyclobutrifluram in *Fusarium fujikuroi*. J. Agric. Food Chem..

